# Suspension culture combined with chemotherapeutic agents for sorting of breast cancer stem cells

**DOI:** 10.1186/1471-2407-8-135

**Published:** 2008-05-14

**Authors:** Hai-zhi Li, Tong-bo Yi, Zheng-yan Wu

**Affiliations:** 1 Department of General Surgery, First Affiliated Hospital, Nanjing Medical University, Nanjing, China

## Abstract

**Background:**

Cancer stem cell (CSC) hypothesis has not been well demonstrated by the lack of the most convincing evidence concerning a single cell capable of giving rise to a tumor. The scarcity in quantity and improper approaches for isolation and purification of CSCs have become the major obstacles for great development in CSCs. Here we adopted suspension culture combined with anticancer regimens as a strategy for screening breast cancer stem cells (BrCSCs). BrCSCs could survive and be highly enriched in non-adherent suspension culture while chemotherapeutic agents could destroy most rapidly dividing cancer cells and spare relatively quiescent BrCSCs.

**Methods:**

TM40D murine breast cancer cells were cultured in serum-free medium. The expression of CD44^+^CD24^- ^was measured by flow cytometry. Cells of passage 10 were treated in combination with anticancer agents pacilitaxel and epirubicin at different peak plasma concentrations for 24 hours, and then maintained under suspension culture. The rate of apoptosis was examined by flow cytometry with Annexin-V fluorescein isothiocyanate (FITC)/propidium iodide (PI) double staining method. Selected cells in different amounts were injected subcutaneously into BALB/C mice to observe tumor formation.

**Results:**

Cells of passage 10 in suspension culture had the highest percentage of CD44^+^CD24^- ^(about 77 percent). A single tumor cell in 0.35 PPC could generate tumors in 3 of 20 BALB/C mice.

**Conclusion:**

Suspension culture combined with anticancer regimens provides an effective means of isolating, culturing and purifying BrCSCs.

## Background

In recent years there has been an increasing focus on the cancer stem cell (CSC) theory. More and more CSCs in solid tumors have been prospectively identified based on expression of cell surface markers [1-5] or Hoechst 33342 dye efflux [6-9]. According to CSC hypothesis, a CSC is a cancer-initiating cell that possesses the ability to recreate a new tumor when transplanted into a suitable microenvironment. However, to date, there have been no studies that convincingly put this proposition into practice. CSCs identified from several solid tumors are only a small subpopulation of tumorigenic cells that most probably represents a heterogeneous mixture of CSCs and their differentiated progeny. More primitive, undifferentiated CSCs (also called true CSCs) remain hidden among them.

Since a single stem cell has been demonstrated to have the ability to reconstitute an entirely functional organ in a mouse model [10-12], a single CSC with stem-like properties should also generate a tumor when injected into a congenetic animal model. It is still unclear whether true CSCs have unique cell surface antigens. To address this basic issue in CSC hypothesis, we explored the possibility of exploiting the feature of drug resistance in cancer cells for screening CSCs on the basis of serum-free suspension culture for isolation and in vitro propagation of tumorigenic cancer cells. By applying suspension culture combined with anticancer regimens, we obtained single breast cancer stem cell (BrCSC), which was able to generate new tumor when injected into congenetic mice.

## Methods

### In vitro propagation of BrCSCs in serum-free culture medium

TM40D murine breast cancer cells were kindly provided by M. Zhang (Baylor College of Medicine, Houston, Texas) and maintained in our laboratory. Cells were cultured in serum-free DMEM/F12 (DrGenes, Shandong, China) containing 20 ng/ml epidermal growth factor (EGF; PeproTech Asia, Rehovot, Israel), 20 ng/ml basic fibroblast growth factor (bFGF; R&D Systems, Minneapolis, MN), 0.4% bovine serum albumin (DrGenes) and 2% B27 (Invitrogen, Carlsbad, CA) at a density of 1000 cells/ml. Cells were grown in T-75 culture flasks at 37°C in a humidified atmosphere containing 5% CO_2_. Tumorspheres were passaged by mechanical dissociation to single cells through the needle (gauge 21) of a 5 ml syringe every 3 days and reseeded in the medium described above at 1000 cells/ml.

### Flow cytometric analysis and apoptosis measurement

To identify CD44^+^/CD24^- ^cells in TM40D cell line, we measured expression of CD44^+^CD24^- ^at passages 2–16 by using a fluorescence-activated cell sorting (FACS)-Vantage SE instrument (Becton Dickinson, Franklin Lakes, NJ). The antibodies used were anti-CD24-phycoerythrin (PE), anti-CD44-PE-cyainn 5 (CY5), and their corresponding isotype controls (BioLegend, San Diego, CA). Cells were harvested and gently disaggregated to a single cell suspension. Staining was made according to the manufacturer's protocol. The rate of apoptosis induced by anticancer regimens was analyzed by flow cytometry using an annexin V-FITC/PI kit (BD Biosciences, San Diego, CA) following the manufacturer's instructions.

### Anticancer regimens screening assay

Cells at passage 10 were plated at 1 × 10^5^/ml into 24-well plates (100 μl per well) under serum-free condition for 3 days until formation of tumorspheres consisting of hundreds of cells. Then a combination with pacilitaxel (Taiji, Sichuan, China) and epirubicin (Pfizer, Wuxi, China), was administered at several different peak plasma concentrations (PPC; 1, 0.5, 0.35, 0,3, 0.25, 0.1). Each concentration was repeated thrice. The PPC of pacilitaxel and epirubicin are 2.2 μg/ml and 0.78 μg/ml, respectively. After incubation for 24 hours, the cells were centrifuged, mechanically dissociated into single cells and recultured for 7 days in fresh serum-free medium.

### Tumor xenograft experiment

As described previously by Al-Hajj et al [1], four-week-old female BALB/C mice (Shanghai Experimental Animal Center, Chinese Academy of Sciences, Shanghai, China) were treated with VP-16 (Double-crane, Beijing, China) by an i.p. injection (30 mg/kg). Five days later, tumor cells injections were done. Cells treated with 0.35 PPC of chemotherapeutic drugs were counted in DMEM/F2 with 0.04% trypan blue (Shenggong, Shanghai, China) and 10% fetal calf serum (Sijiqing, Hangzhou, China), and resuspended in DMEM/F2 and Matrigel (BD Biosciences) mixture (1:1 volume). The cells were then injected s.c. in 200 μl volumes into the abdominal wall of the mice. Cell viability was determined by trypan blue exclusion before transplantation. The number of injected cells was obtained with limited dilution method. After cell injection, mice received an estradiol (Tongyong, Shanghai, China) supplementation (0.4 mg/kg s.c.) every 10 days for 40 days and were inspected for tumor appearance by observation and palpation as described by Ponti et al. [19]. When the xenograft tumors grew to about 1 cm in diameter, the mice were sacrificed by cervical dislocation and each tumor was confirmed by routine H&E staining for paraffin sections. Eight months later, the others that failed to give rise to palpable nodules at the injection site were also sacrificed and soft tissues around the injection site were examined by H&E staining. Experimental protocols were performed according to the guidelines of the Nanjing Medical University Animal Committee.

### Expression of CD44 and CD24 in xenograft tumors by immunohistochemical analysis

Following the diagnosis of breast cancer by H&E staining, immunohistochemical procedures were made on 4-μm tissue sections with monoclonal antibodies for CD44 and CD24 (both from Boster, Wuhan, China). Immunodetection was performed using a standard SABC method. Sections were counterstained blue with hematoxylin for the nuclei.

## Results

### Enrichment of the subpopulation of putative BrCSCs in TM40D cells under suspension conditions

**Figure 1:**
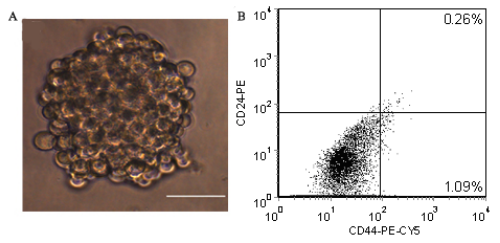
**(A) floating tumorspheres after 6 days of suspension culture.** Scale bar = 100 μm. (B) CD44^+^CD24^- ^expression in TM40D cell line.

**Figure 2:**
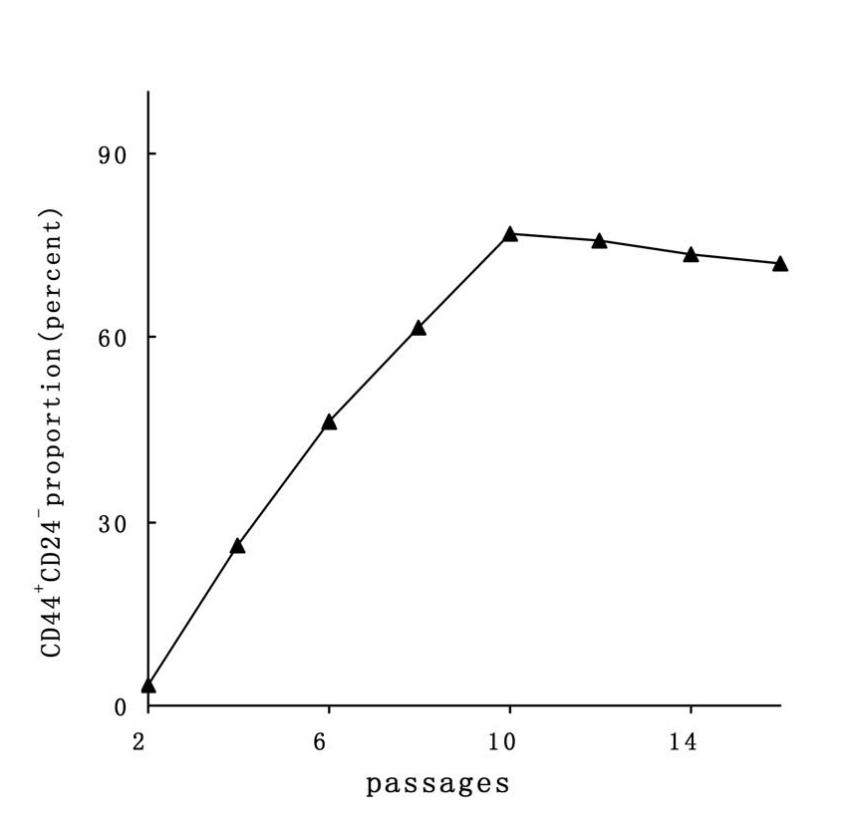
CD44^+^CD24^- ^expression under serum-free culture condition during serial passages.

TM40D murine breast cancer cells could be directly adapted from serum-containing medium to serum-free medium. Primary tumorspheres were initially observed at the fifth day of serum-free culture. Large spherical aggregates consisting of hundreds of cells, varying from 60 to 200 μm in diameter, formed after 7 days of culture (Fig. 1A). Tumorspheres were then disaggregated into a single cell suspension and serially passaged at clonal density (1000 cells/ml). Secondary tumorspheres appeared on the second day of culture. To determine the proportion of BrCSCs in TM40D cells under suspension conditions and obtain sufficient BrCSCs in quantity, we measured expression of CD44^+^CD24^- ^during serial passages. The CD44^+^CD24^- ^subpopulation in TM40D cell line accounted for 0.7 – 1.6% in serum-containing medium (Fig. 1B). The percentage of CD44^+^CD24^-^cells had a marked increase at passages 2 to 10 (from 3.39% up to 72.42%), and then it maintained a relatively stable proportion (Fig. 2).

### BrCSCs sorted by suspension culture combined with chemotherapeutic agents

Cells of the 10th passage under suspension culture were incubated in combination with different concentrations of pacilitaxel and epirubicin for 24 hours. The cells were then washed thoroughly with HBSS to remove chemotherapy drugs and maintained in suspension culture by gently agitating aggregates as single cells. By centrifugaion to exclude dead cells and debris, fresh culture media were replaced every 3 days. After 7 days of culture in serum-free medium, cell apoptosis was evaluated by flow cytometry with Annexin-V FITC/PI double staining method. As shown in Table 1, all cells treated with 1 and 0.5 PPC of pacilitaxel and epirubicin, i.e. TE chemotherapy protocol, underwent apoptosis and necrosis. Cells treated with 0.25 and 0.1 PPC of TE survived, proliferated, and formed tumorspheres. The vast majority of cells treated with 0.35 PPC of TE (~98%) suffered death.

**Table 1:** Apoptosis and necrosis induced by combination with pacilitaxel and epirubicin

Percentage of FACS events	PPC of pacilitaxel and epirubicin					
	
	1	0.5	0.35	0.3	0.25	0.1
Apoptosis rate	100	100	98.28 ± 1.43	81.23 ± 3.56	-	-

NOTE: not detection in 0.25 and 0.1 PPC of PE groups due to cell proliferation and formation of tumorspheres.

### Self-renewal and differentiation of BrCSCs in vitro

**Figure 3:**
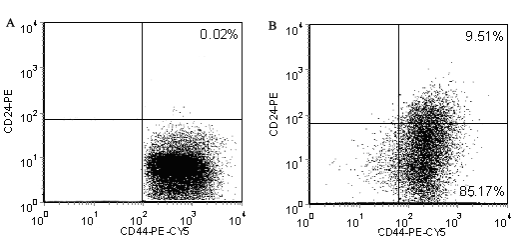
**CD44^+^CD24^- ^expression in cells treated with 0.35PPC of PE.** (A) one week after chemotherapy. (B) two weeks after chemotherapy.

As BrCSCs have been identified as CD44^+^CD24^- ^phenotypes, we measured the expression of cells treated with 0.35 PPC of TE one week after chemotherapy. During this period, no tumorspheres and cell proliferation were observed under the microscope. The vast majority of cells (>99.8%) were positively for CD44 and negatively for CD24 (Fig. 3A). Then these cells were isolated and plated in 96-well culture dishes at a density of 1 cell per well under serum-free media. After another week, clusters of cells were found in more than 90% of the wells. The tumorspheres were collected and dissociated as described above, and the expression of CD44 and CD24 were evaluated again using flow cytometry. About 87% of cells stained CD44^+^CD24^- ^(Fig. 3B).

### Tumor reconstitution by a single breast cancer cell

**Figure 4:**
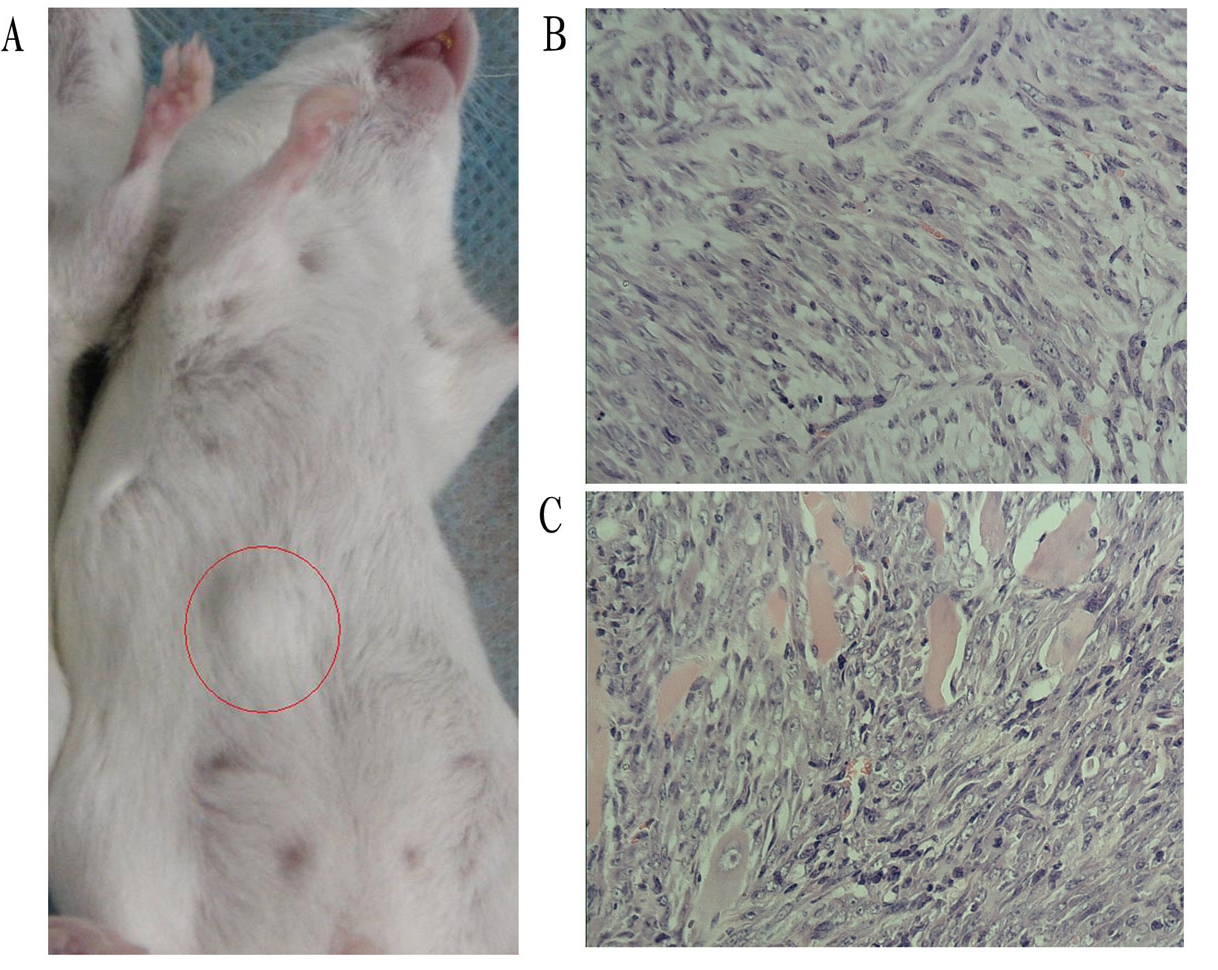
**(A) a visible tumor driven by a single cell in a mouse with normal immune function.** (B) a pathological section of tumor tissues from 10^6 ^TM40D cells transplantation. (C) a pathological section of tumor tissues from a single breast cancer cell transplantation. (both 20 objective).

**Figure 5:**
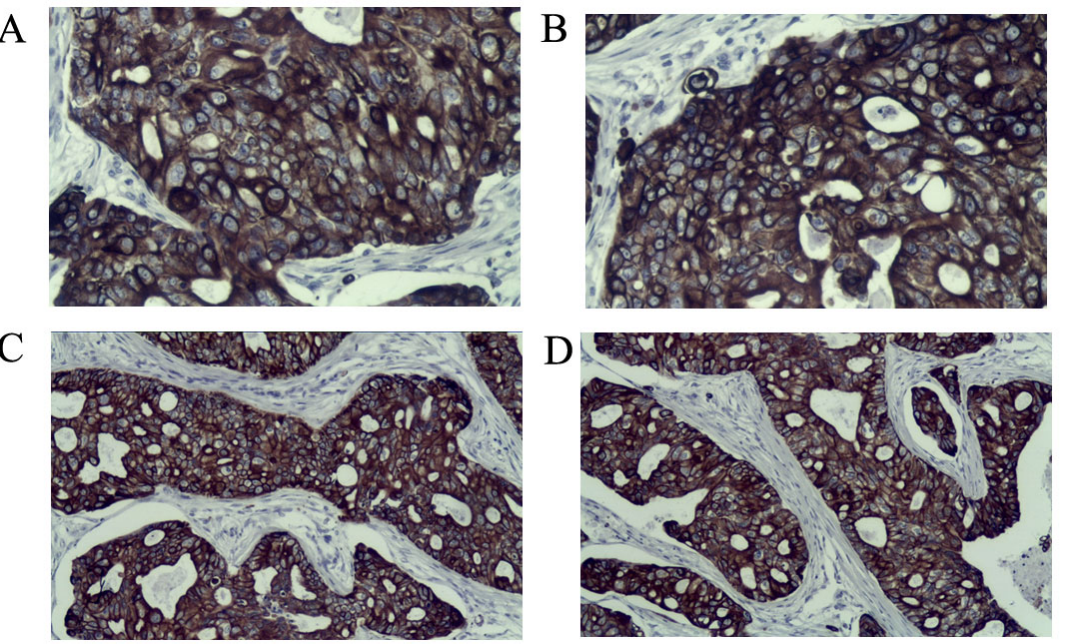
**The expression of CD44 and CD24 of histological sections by immunochemistry analysis.** (A, B) CD44 and CD24 staining from the xenograft tumors driven by a single sorted cell, respectively. (C, D) CD44 and CD24 staining from the xenograft tumors driven by unsorted TM40D cells, respectively. (both 20 objective).

To test tumorigenic ability of the sorted cells, cells which survived 0.35 PPC of chemotherapeutic agents were injected into the abdominal wall of BALB/C mice, and CD44^+^CD24^+ ^cells and CD44^+^CD24^- ^cells from the passage 10 under suspension culture were used as the control group. One month after injection, tumors could be observed at the injection sites of 1000 and 100 cells treated with 0.35 PPC of TE in all mice with normal or suppressed immune function. 1000 and 100 CD44^+^CD24^- ^cells gave rise to tumors in most cases. By contrast, 1000 or 100 CD44^+^CD24^+^cells failed to form tumors (Table 2). After about 10 weeks, a single cell of 0.35 PPC of TE group reconstituted an entire tumor in 3 of 20 mice, one with normal immune function, the other two with suppressed immune function (Fig. 4A). Compared with previous tumor sections from TM40D cells, tumors formed by a single sorted cell displayed the same histological appearance by hematoxylin and eosin staining paraffin sections (Fig. 4B and 4C). The expression of CD44 and CD24 could be found in the sections from the xenograft tumors driven by a single sorted cell, and had no significant differences compared with sections from TM40D cells (Fig. 5). Eight months after tumor inoculation, normal mouse tissue was observed at the injection sites that failed to give rise to form palpable nodules by histology.

**Table 2:** Engraftment of murine breast cancer cells into BALB/C mice

	Tumors/injections		
	
	1000	100	1
Mice with normal immune function			
Cells treated with 0.35 PPC of TE	7/7	7/7	1/10
CD44^+^CD24^+ ^cells	0/7	0/7	-
CD44^+^CD24^- ^cells	7/7	5/7	0/7
Mice with suppressed immune function			
Cells treated with 0.35 PPC of TE	7/7	7/7	2/10
CD44^+^CD24^+ ^cells	0/7	0/7	-
CD44^+^CD24^- ^cells	7/7	7/7	0/7

## Discussion

Since BrCSCs have been identified as CD44^+^CD24^- ^subpopulation from breast cancer patient samples [1], much progress in research on this field has been achieved. However, the isolation and purification of BrCSCs is still hindered by their scarcity in quantity and continuous differentiation. To the development of targeted therapy against breast cancer, the key is to solve the problem from the identification of true BrCSCs. At present, identification of CSCs is mainly based on cell surface antigens sorted by fluorescence-activated cell sorting or magnetic immunosorting. As the exact molecular signature of CSCs is not known, it seems somewhat blind and impractical in order to find CSCs by screening large numbers of known markers. Hoechst 33342 dye efflux, another sorting approach in using functional properties of CSCs, are being utilized to isolate side population cells enriched in CSCs. However, cytotoxicity of Hoechst-33342 may restrict its further applications [13-15]. The above two methods both use flow cytometry, and cell sorting may cause the trauma of stained cells.

The CSC theory holds that CSCs reside in a largely quiescent state with regard to cell-cycle activity [16,17] and express high levels of ATP-binding cassette (ABC) transporters such as the multidrug resistance gene [18], so that they can be more resistant to chemotherapeutics than tumour cells with limited proliferative potential. Furthermore, it has been demonstrated that BrCSCs can be propagated and passaged in vitro under serum-free suspension culture and sustain the undifferentiated state [19]. Based on the above theory and research results, we propose a novel strategy-suspension culture combined with anticancer regimens – to screen BrCSCs. At first, we followed the approach of Ponti et al. [19] to expand and enrich tumorigenic breast cancer cells under suspension culture conditions. Cytotoxic regimens were then used to eradicate rapidly dividing tumor cells but spare BrCSCs. Finally, tumor xenograft experiments were performed to evaluate tumorigenicity of the sorted cells. Recently, Yu et al. [20] have successfully taken advantage of mammospheres in suspension culture and chemotherapy to enrich for self-renewing breast cancer cells, which further confirms our viewpoint.

Tumorspheres could sustain long-term suspension culture and serial passage in vitro. In our laboratory, they have been through 110 successive passages. During the period of serum-free culture, we observed that cells were not able to proliferate in serum-free medium without EGF and/or bFGF. Tumorspheres only formed in the presence of EGF and/or bFGF. Similar results have also been in culture of mammospheres reported by Dontu et al.[21]. We speculate it is EGF and/or bFGF that induce cell proliferation under suspension culture conditions. As described by Ponti et al. [19] and Yu et al.[20], tumorspheres were undifferentiated cells because they failed to express mature markers associated to myoepithelial cells (cytokeratin 14 and alpha-smooth muscle actin) and luminal/ductal cells (cytokeratin 14 and MUC-1). However, it is the relative degree of undifferentiation. In this experiment we found that the sorted CD44^+^CD24^- ^subset could generate CD44^-^CD24^-^, CD44^-^CD24^+^, and CD44^+^CD24^+ ^subpopulation besides CD44^+^CD24^- ^phenotype in serum-free culture (data not shown). Even if under suspension culture, tumorsphere cells propagate always followed by differentiation. Before chemotherapy, more than 70% of cells in serum-free media possessed CD44^+^CD24^- ^phenotype. One week after chemotherapy, almost all cells expressed CD44^+^CD24^-^.(Note early flow cytometry analysis after anticancer application may affect test results as a large number of necrotic cells and debris). The findings showed that chemotherapy drugs killed non-CD44^+^CD24^- ^cells and only retained a very small number of CD44^+^CD24^- ^cells in 0.35 PPC of TE (less than 2%). After about 2 weeks of quiescence and recovery, a single surviving CD44^+^CD24^-^cell would propagate as tumorspheres and differentiate into a heterogenous population. These cells exhibted the ability of self-renewal and multipotent differentiation in virto suspension culture.

In order to obtain a true, single BrCSC, we have proposed in vitro suspension culture growth pattern to describe the properties of BrCSCs from suspension culture in vitro [22]. Tumorspheres consist of BrCSCs and transit amplifying cells/progenitors, which may be compared to the structure of an atomic model. A few BrCSCs locate inside the core of tumorsphere (viewed as an atomic nucleus) and a large amount of relatively differentiated progenies surround the outer layer (viewed as electrons). BrCSCs simultaneously perpetuate themselves through symmetric division and generate differentiated progeny through asymmetric division [23,24]. Self-renewal of BrCSCs is limit and tumorpsphere formation is mainly due to expansion of their progeny (rapidly dividing cells, also termed transit amplifying cells). A possible mechanism may involve that BrCSCs have a relatively slow turnover rate to ensure uniform genetic background and rapid expansion of transit amplifying cells prevent neighboring BrCSCs proliferating via unknown regulation. As gentle mechanical aspiration can disperse tumorspheres, surface tension may play a key role in tumorpsphere formation rather than cell-cell interactions. From this hypothesis model, we can speculate that a certain size tumorsphere should contain the largest proportion of BrCSCs. To obtain a large number of BrCSCs, it is necessary to dissociate the optimal size of tumorspheres into single cells. In this way, single BrCSC is exposed again and another self-renewal process will continue. By measuring CD44^+^CD24^- ^expression of tumorsphere cells during serial passages, we found that it had the highest proportion of CD44^+^CD24^- ^cells at passage 10. Cells of passage 10 were therefore the most desirable research subjects. As shown in Fig. 2, the proportion of CD44^+^CD24^-^subpopulation exhibited a rough S-shaped curve. This suggests that CD44^+^CD24^- ^cells has achieved stable proportion after some passages of suspension culture and BrCSCs may reside in a relatively slow proliferation period or a quiescent state, whereas their differentiated progeny may sustain expansion in number. During this time, most BrCSCs can survive the attack of chemotherapy drugs. Pacilitaxel and epirubicin, which mainly target rapidly dividing tumor cells, are widely used against breast cancer as a combined chemotherapy protocol. It is a critical issue to determine the optimal concentrations of TE. The criteria is required to comply with the requirements of eradicating the vast majority of dividing cells and retaining the minimum number of dormant cells. Less than 2% of the cells treated with 0.35 PPC of TE survived the double sorting of suspension culture and anticancer regimens. In theory, these sorted cells were much closer to the source of primitive BrCSCs. In contrast, the other concertrations of chemotherapy drugs would kill all tumor cells or would not stop cell proliferation in our study.

Tumorigenicity test are currently been regarded as the gold standard for proving the existence of CSCs. The key to a successful transplantation is to keep cell viability. Before single-cell inoculation, each cell was evaluated by trypan blue staining to exclude dead cells. At the same time matrigel and estrogen were both applied. Matrigel can provide a more favourable microenvironment niche for cell colonization and growth. Exogenous estrogen supplementation can promote tumor formation. In this study, in order to avoid transplantation rejection in the use of human breast cancer cells and immunocompromised mice, we adopted the established breast cancer cell line TM40D from BALB/C mice. Although congenic transplantations were used, we applied VP-16 before transplantation in order to reduce immune and inflammation response and guarantee the viability of cells after inoculation under the consideration of transplantation of a small number of tumor cells at initial design stage, especially for a single-cell transplantation. In fact, the transplantation results showed there were no distinct difference between the non-immunosupressed and immunosuppressed group in this study. It suggests that immune function may have no apparent effect on syngeneic tumor transplantation of inbred mice. The immunochemical analysis of the resulting tumors driven by a single sorted cell showed a heterogenous tumor in vivo, with the similar features compared with primary tumors from TM40D cells. Taken in vitro and in vivo experimental studies together, we conclude that the sorted cells are true BrCSCs.

As a functional sorting method, suspension culture combined with anticancer regimens provides us a novel approach to enrich and purify CSCs. At the pre-experiment, we adopted CD29 and CD24 as the presumed markers of BrCSCs according to CD29^hi^CD24^+ ^from mouse mammary epithelium stem cell identified by Shackleton et al. [12]. We found no tumors were formed by injecting 10^4 ^CD29^hi^CD24^+ ^cells or CD29^hi^CD24^- ^into BALB/C mice. The result reminded us that there may be unknown or no specific cell surface antigens highly expressed in BrCSCs. So we bagen to enrich tumorgenic breast cancer cells by serum-free suspension culture and purify BrCSCs by chemotherapy drugs. The results showed BrCSCs in mice derived from CD44^+^CD24^- ^subpopulation and had the same markers of puative BrCSCs in human. By applying this sorting stragegy, we could expand and purify CSCs under the condition of unknown putative markers in vitro. Similarly, we could also obtain CSCs much closer to the source under the condition of known putative markers. It could not be made only by flow sorting of the original cultures.

## Conclusion

In this study, we identified BrCSCs by suspension culture combined with anticancer regimens and confirmed that a single BrCSC can form breast cancer in mice. We showed for the first time, to our knowledge, that a single tumor cell can repopulate a new tumor in mice with normal immune function. The sorting method of suspension culture combined with anticancer regimens for BrCSCs may be applied to identify other CSCs in solid tumors.

## Abbreviations

CSCs – cancer stem cell, BrCSCs – breast cancer stem cells, EGF – epidermal growth factor, bFGF – basic fibroblast growth factor, PE – phycoerythrin, CY5 – cyanin 5, FITC – fluorescein isothiocyanate, PI – propidium iodide, PPC – peak plasma concentration, FACS – fluorescence-activated cell sorting.

## Competing interests

The authors declare that they have no competing interests.

## Authors' contributions

Most experiments and the manuscript were made by H–ZL. T–BY performed immunocytochemistry staining. Z–YW was involved in designing all experiments and revised the manuscript. All authors read and approved the final manuscript.

## Pre-publication history

The pre-publication history for this paper can be accessed here:



## References

[B1] Al-Hajj M, Wicha MS, Benito-Hernandez A, Morrison SJ, Clarke MF (2003). Prospective identification of tumorigenic breast cancer cells. Proc Natl Acad Sci USA.

[B2] Singh SK, Clarke ID, Terasaki M, Bonn VE, Hawkins C, Squire J, Dirks PB (2003). Identification of a cancer stem cell in human brain tumors. Cancer Res.

[B3] Collins AT, Berry PA, Hyde C, Stower MJ, Maitland NJ (2005). Prospective identification of tumorigenic prostate cancer stem cells. Cancer Res.

[B4] O'Brien CA, Pollett A, Gallinger S, Dick JE (2007). A human colon cancer cell capable of initiating tumour growth in immunodeficient mice. Nature.

[B5] Li C, Heidt DG, Dalerba P, Burant CF, Zhang L, Adsay V, Wicha M, Clarke MF, Simeone DM (2007). Identification of pancreatic cancer stem cells. Cancer Res.

[B6] Hirschmann-Jax C, Foster AE, Wulf GG, Weidner D, Marini F, Brenner MK, Andreeff M, Goodell MA (2004). A distinct 'side population' of cells with high drug efflux capacity in human tumor cells. Proc Natl Acad Sci USA.

[B7] Szotek PP, Pieretti-Vanmarcke R, Masiakos PT, Dinulescu DM, Connolly D, Foster R, Dombkowski D, Preffer F, Maclaughlin DT, Donahoe PK (2006). Ovarian cancer side population defines cells with stem cell-like characteristics and Mullerian Inhibiting Substance responsiveness. Proc Natl Acad Sci USA.

[B8] Chiba T, Kita K, Zheng YW, Yokosuka O, Saisho H, Iwama A, Nakauchi H, Taniguchi H (2006). Side population purified from hepatocellular carcinoma cells harbors cancer stem cell-like properties. Hepatology.

[B9] Ho MM, Ng AV, Lam S, Hung JY (2007). Side population in human lung cancer cell lines and tumors is enriched with stem-like cancer cells. Cancer Res.

[B10] Osawa M, Hanada K, Hamada H, Nakauchi H (1996). Long-term lymphohematopoietic reconstitution by a single CD34-low/negative hematopoietic stem cell. Science.

[B11] Krause DS, Theise ND, Collector MI, Henegariu O, Hwang S, Gardner R, Neutzel S, Sharkis SJ (2001). Multi-organ, multi-lineage engraftment by a single bone marrow-derived stem cell. Cell.

[B12] Shackleton M, Vaillant F, Simpson KJ, Stingl J, Smyth GK, Asselin-Labat ML, Wu L, Lindeman GJ, Visvader JE (2006). Generation of a functional mammary gland from a single stem cell. Nature.

[B13] Siemann DW, Keng PC (1986). Cell cycle specific toxicity of the Hoechst 33342 stain in untreated or irradiated murine tumor cells. Cancer Res.

[B14] Chen AY, Yu C, Bodley A, Mathew TL (1993). A new mammalian DNA topoisomerase I poison Hoechst 33342: cytotoxicity and drug resistance in human cell cultures. Cancer Res.

[B15] Singh S, Dwarakanath BS, Mathew TL (2004). DNA ligand Hoechst-33342 enhances UV induced cytotoxicity in human glioma cell lines. J Photochem Photobiol B.

[B16] Guan Y, Gerhard B, Hogge DE (2003). Detection, isolation, and stimulation of quiescent primitive leukemic progenitor cells from patients with acute myeloid leukemia(AML). Blood.

[B17] Holyoake T, Jiang X, Eaves C, Eaves A (1999). Isolation of a highly quiescent subpopulation of primitive leukemic cells in chronic myeloid leukemia. Blood.

[B18] Zhou S, Schuetz JD, Bunting KD, Colapietro AM, Sampath J, Morris JJ, Lagutina I, Grosveld GC, Osawa M, Nakauchi H, Sorrentino BP (2001). The ABC transporter Bcrp1/ABCG2 is expressed in a wide variety of stem cells and is a molecular determinant of the side-population phenotype. Nat Med.

[B19] Ponti D, Costa A, Zaffaroni N, Pratesi G, Petrangolini G, Coradini D, Pilotti S, Pierotti MA, Daidone MG (2005). Isolation and in vitro propagation of tumorigenic breast cancer cells with stem/progenitor cell properties. Cancer Res.

[B20] Yu F, Yao H, Zhu P, Zhang X, Pan Q, Gong C, Huang Y, Hu X, Su F, Lieberman J, Song E (2007). let-7 regulates self renewal and tumorigenicity of breast cancer cells. Cell.

[B21] Dontu G, Abdallah W, Foley J, Jackson KW, Clarke MF, Kawamura MJ, Wicha MS (2003). In vitro propagation and transcriptional profiling of human mammary stem/progenitor cells. Gene Dev.

[B22] Li HZ, Yi TB, Wu ZY (2007). Suspension culture combined with anticancer regimensfor screening breast cancer stem cells. Med Hypotheses.

[B23] Boman BM, Wicha MS, Fields JZ, Runquist OA (2007). Symmetric Division of Cancer Stem Cells – a Key Mechanism in Tumor Growth that should be Targeted in Future Therapeutic Approaches. Clin Pharmacol Ther.

[B24] Morrison SJ, Kimble J (2006). Asymmetric and symmetric stem-cell divisions in development and cancer. Nature.

